# Effect of Antibiotic Prophylaxis in Dental Implant Surgery: A Randomized Controlled Clinical Trial

**DOI:** 10.3390/dj13110500

**Published:** 2025-10-28

**Authors:** Fernando Bravo-Olmedo, Candela Reyes-Botella, Francisco Manuel Ocaña-Peinado, Francisco Javier Manzano-Moreno, Maria de Nuria Romero-Olid, Maria Victoria Olmedo-Gaya

**Affiliations:** 1 School of Dentistry, University of Granada, 18071 Granada, Spain; ferbravo@correo.ugr.es (F.B.-O.); creyes@ugr.es (C.R.-B.); nromero@ugr.es (M.d.N.R.-O.); mvolmedo@ugr.es (M.V.O.-G.); 2 Department of Stomatology, School of Dentistry, University of Granada, 18071 Granada, Spain; 3 Biomedical Group (BIO277), University of Granada, 18071 Granada, Spain; 4 Instituto Investigación Biosanitaria, ibs.Granada, 18071 Granada, Spain; 5 Department of Statistics and Operations Research, University of Granada, 18071 Granada, Spain; fmocan@ugr.es

**Keywords:** amoxicillin, antibiotic prophylaxis, antibiotic resistance, dental implants, early failure, infection, RCT

## Abstract

**Background**: The problem of antibiotic resistance is becoming increasingly serious worldwide due to uncontrolled prescription of antibiotics. Studies show conflicting results on the use or not of antibiotic prophylaxis associated with dental implant placement; its benefits are unclear, and its use is increasingly questioned. The aim of this randomized controlled clinical trial (RCT) was to compare early implant failure and postoperative infectious complications between two groups of healthy, non-penicillin-allergic patients who received a single prophylactic dose of 2 g amoxicillin versus placebo 1 h before surgery for implants placed in a single operative field. **Methods:** A double-blind, parallel-group, single-center RCT was conducted. One hundred patients met the inclusion criteria and were randomly assigned to the amoxicillin (*n* = 50) or placebo (*n* = 50) group. The primary endpoints analyzed were early implant failure and the presence of postoperative infection at 7, 14, 30 and 90 days. The recommendations of the CONSORT 2025 statement for RCT reporting were followed. **Results:** A total of 151 implants were placed in 96 patients and 12 implants failed; 6 implants in the antibiotic group (7.7%) and 6 implants in the placebo group (8.2%), so no statistically significant differences were observed between groups in the rate of early implant failure. In contrast, 11 implants developed postoperative infection; 2 in the antibiotic group (2.6%) and 9 in the placebo group (12.3%), reaching statistically significant differences (*p* = 0.028). **Conclusions:** The use of antibiotic prophylaxis in healthy patients is not necessary to prevent early failure of implants placed in a single operative field; however, the higher rate of infectious complications in patients without antibiotic therapy still raises a question that requires further investigation.

## 1. Introduction

Dental implant treatment is currently the most common surgical procedure performed by dental professionals. According to recent estimates, more than 20 million implants are placed worldwide. Current systematic reviews find a 10-year success rate of these procedures of 95.2–97.5% [1,2,3]. Dental implant failures are classified into early failure and late failure, depending on whether they occur before or after placement of the final prosthesis [2]. Early dental implant failure is defined as a loss of the implant that occurs before the implant has successfully integrated with the jawbone, or within the initial months after placement, often before a final restoration is attached [3,4]. Most failures occur during the osseointegration phase, accounting for 83% of all failures, and are due to bacterial contamination at the implant insertion site, among other factors such as a lack of primary stability, tobacco smoking, non-controlled periodontitis, poor bone quality, or implants that are too short [5,6,7].

In order to reduce the rate of early implant failure and taking into account that the oral cavity has more than 600 bacterial strains that can contribute to contamination and loss of osseointegration, different antibiotic guidelines included in recent reviews and meta-analyses [3,5] have been proposed to limit the occurrence of postoperative infection. These studies have found a reduction in early implant failure in healthy patients given a single prior antibiotic prophylaxis. There is controversy in its use due to the risk of indiscriminate antibiotic administration to systemically healthy subjects, which may trigger adverse reactions and/or the development of bacterial resistance [6,7].

In the field of oral surgery, antibiotic prophylaxis is only recommended in patients at risk of endocarditis, immunocompromised patients, when surgery is performed in areas with infection, for extensive and prolonged surgeries, and when immediate implants are placed or bone regeneration techniques are performed [8,9]. Considering that the main cause of early failure is the presence of infection, in order to prevent this early failure associated with bacterial contamination, different antibiotic therapies have been proposed pre- and post-implant surgery in systemically healthy individuals [10,11]. Microbiological control through the use of antibiotic prophylaxis seems logical to allow a more aseptic environment during implant placement, favoring bone tissue healing and osseointegration [12]. The antibiotic most commonly used in clinical trials has been amoxicillin. Initially, different regimens were tested: some combined previous therapy with several days of additional treatment, while others only prolonged therapy before or after surgery. However, there is still some controversy, although it is known that the use of an antibiotic regimen helps prevent early implant failure [3].

On the one hand, the cost-effectiveness from the patient’s point of view is positive, even though it increases the cost of the antibiotic since it decreases the failure rate, making the treatment more effective according to a recently published study [13]. But at the other extreme, indiscriminate antibiotic intake in healthy subjects can lead to the development of antibiotic-resistant bacteria [14], affecting public health and associated costs [13].

The aim of the present randomized controlled clinical trial (RCT) was to compare the effect of 2 g amoxicillin taken 1 h preoperatively on early implant failure and postoperative infections in American Society of Anesthesiologists (ASA) I and ASA II patients who had implants placed in a single operative field. The null hypothesis was that there is no difference in early failure and postoperative infections between patients receiving antibiotic prophylaxis and those not receiving antibiotic prophylaxis. The alternative hypothesis was that there is significative difference in early failure and postoperative infections between patients receiving antibiotic prophylaxis and those not receiving antibiotic prophylaxis.

## 2. Materials and Methods

### 2.1. Study Design

A single-center double-blinded RCT with parallel groups was undertaken in patients treated at the Clinic of the Masters in Oral Surgery and Implantology of the University of Granada from October 2023 to December 2024. The trial was approved by the Human Research Ethics Committee of the University of Granada (4034/CEIH/2024) and registered in the Clinical Trial Registry of Australia and New Zealand (ANZCTR; No.ACTRN12624001151527), and the recommendations of the CONSORT 2025 statement (Table S1) for RCT reporting were followed [15].

### 2.2. Patient Selection

Inclusion criteria were: age ≥ 18 years, need for implant placement in a single operative field (1–6 implants), ASA I/ASA II patients, absence or control of periodontal disease and presence of sufficient bone and soft tissue not involving augmentation surgery at the time of placement. Exclusion criteria were: patients whose medical condition required postoperative antibiotic prophylaxis (e.g., immunosuppressed, etc.), patients who underwent head and neck radiotherapy less than two years ago, presence of uncontrolled diabetes, untreated periodontitis, allergic to penicillin, patients treated with antiresorptive drugs, pregnant or lactating women, patients taking antibiotics for other reasons (up to one month before implant surgery), immediate post-extraction implants and implants requiring simultaneous bone regeneration at the time of placement (Table 1).

All participants signed informed consent to participate in the study, which complied with the ethical principles of the Helsinki Declaration. The sample size determination was based on detecting a clinically relevant minimum difference of 6 percentage points (7% implant failure in the control group versus 1% in the antibiotic group) in accordance with the guidelines proposed by Wittes [16], as well as similar previous publications on the use of antibiotics in implant dentistry [17,18]. Using a two-sided significance level of 0.05 (Type I error rate) and a statistical power of 80% (Type II error rate of 0.20), the calculation (based on a Z-test for two independent proportions) yielded a minimum required sample size of 90 evaluable patients. This value was increased by 10% (resulting in 100 patients, 50 per group) to account for potential dropouts and losses to follow-up. Patients were consecutively admitted to the study and randomized using a computer-generated sequence to one of the two study groups. Each participant was assigned (1:1 ratio) a random code (A or B), with code A representing the amoxicillin 2 g group and patients took 2 tablets of 1 g (Laboratorios Cinfa SA, Pamplona, Navarra) and code B representing placebo, with patients taking 2 tablets, in both cases 1 h before the intervention. The placebo was prepared by the pharmacy in tablets identical in size, color and shape to the 1 g amoxicillin to ensure blinding. All data were collected by the principal investigator (MV.O.G), who was unaware of the assignment of patients to treatment groups, and neither the patients nor the operator were aware of the treatment administered to the patient. Finally, the statistics consultant was also unaware of the group to which patients belonged until completion of the study.

### 2.3. Surgical Protocol

All interventions were performed by the same postgraduate student of the Master’s in Oral Surgery and Implantology (F.O.B). The implant system used in the patients was the Shelta implant^®^ (Sweden & Martina, Due Carrare, Italy). Prior to surgery, the patient performed a mouth rinse with 0.12% chlorhexidine (CHX) and 0.05% CPC (Perio-Aid^®^, Dentaid, Barcelona, Spain). The perioral area was then disinfected using sterile gauze soaked in CHX. Subsequently, the patient’s head, eyes and torso were covered with sterile surgical drapes to maintain an aseptic environment. After local anesthesia with 4% articaine with 1:100.00 epinephrine (Ultracain, Normon, Madrid, Spain), a crestal or paracrestal incision with or without releasing incision was made using a scalpel with a 15C blade. If necessary, the incision was widened by unloading to ensure proper exposure. This step facilitated complete visualization of the operative area, essential for reaming and accurate implant placement. To detach the flap, a periosteal flap was used, moving first in the vestibular area and then in the palatal or lingual area, allowing optimal visibility of the surgical site. Using a surgical micromotor (Surgic Pro, NSK^®^, Kanuma, Japan) and constant irrigation with saline, the implant bed was prepared according to the drilling sequence indicated by the manufacturer, depending on the bone quality and diameter of the implant to be placed. The implants were inserted with a torque between 20 and 40 Ncm, and the cover screw was placed to protect the implant during the osseointegration process. Finally, the wound was sutured using 5/0 non-resorbable polyamide (Seralon; Osteogenos, Madrid, Spain). Both oral and written instructions on postoperative care were provided. Regarding postoperative pain management, administration of 400 mg ibuprofen or 1 g paracetamol every 8 h was recommended as long as necessary. The patient’s data collection sheet was provided, where a daily assessment of pain and swelling during the first week was recorded, using the Visual Analogue Scale (VAS) as well as the number of tablets taken each day to control postoperative pain and swelling. Patients were instructed to use 0.12% chlorhexidine gel + hyaluronic acid three times a day for 14 days.

The postoperative follow-up protocol was designed to evaluate the patient at 7, 14, 30 and 90 days after implant placement. In case of postoperative infection, amoxicillin 750 mg was administered every 8 h for 7 days. At the final check-up at day 90, signs of infection at the implant site were assessed first, followed by the second phase.

### 2.4. Study Outcomes

Study outcomes were classified as primary and secondary outcomes. The primary outcome was *early implant failure,* defined as loss of the implant before prosthetic loading [3,4]. The timing of implant loss was also assessed.

Secondary outcomes were: *infectious complications* identified by the presence of severe pain after the first 48–72 h, swelling from postoperative day 7, fever greater than 38°, warmth, discharge (fistula) and/or erythema. The occurrence of infectious complications at 7, 14, 30 and 90 days was also assessed, as well as *Postoperative pain and swelling* during the first 7 days after surgery using a VAS (0 = no pain/swelling and 10 = maximum pain and swelling imaginable), with recordings of the number of analgesic/anti-inflammatory medication tablets required during the first 7 days. In addition, any adverse events (e.g., nausea, vomiting, diarrhea, exanthema, dyspepsia, or acidity) were recorded.

The other variables collected were the patient variables; age, sex, general health (American Society of Anesthesiologists [ASA] I or ASA II), systemic medication (yes/no), history of periodontitis (yes/no), smoker (no/<10 cig/>10 cig), number of implant (1/2–3/4–6), implant location (maxilla/mandible), replacing tooth and implant bed bone quality (type I, II, III or IV in the Lekholm & Zarb classification) [19].

For the implant variables, implant length (<8.5/> or =8.5 mm) and diameter (3.3 mm/3.8–4.25 mm/5 mm) were collected.

Finally, the type of incision (linear or linear with releasing incision), use of special expansion/condensation techniques (yes/no), biological reaming (yes/no) and atraumatic sinus elevation (yes/no), duration of surgery (in minutes) were recorded.

### 2.5. Statistical Analysis

Statistical analysis was performed using SPSS v 30.0 (IBM Corp, 2024, Armonk, NY, USA). The normality of the variables was tested using the Shapiro–Wilk test. For all tests performed, the significance level, α, was set at α = 0.05.

We describe the patients with a descriptive analysis, and secondly, a statistical inference analysis was derived. In the descriptive summary, qualitative variables were described using frequencies and percentages. For quantitative variables, the summary was carried out using the mean and standard deviation. For the comparison of quantitative variables, the Mann–Whitney test and the Student *t*-test were used. Homogeneity between samples was tested using the Levene test. The analysis of association between qualitative variables was carried out using Fisher’s exact test or the χ2 test as appropriate.

Analyzing the implants, we used Generalized Estimating Equations (GEEs). This approach was chosen to consider the clustering of multiple implants within individual patients, thereby addressing the intra-patient correlation and the variable number of implants per patient. A binomial distribution with a logit link function was specified for both models. Bivariate GEE analyses and Wald IC intervals for OR were performed to examine the effect of each predictor on the outcomes.

## 3. Results

### 3.1. Patient Characteristics and Interventions

**Figure 1:**
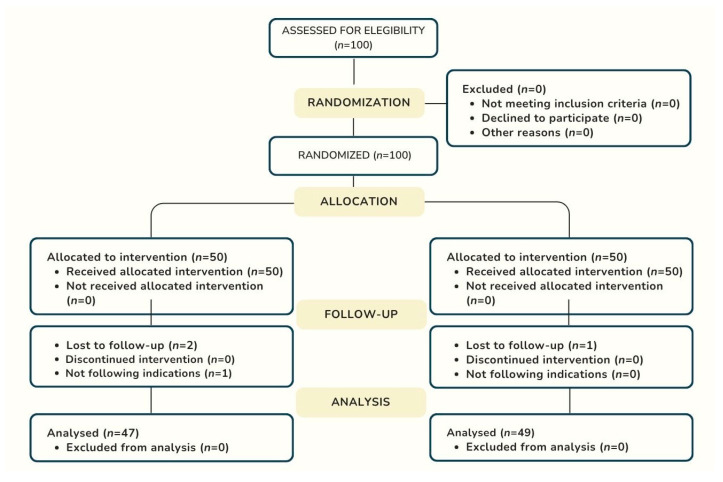
Flowchart of the enrollment process according to Consort 2025 Guideline.

One hundred patients who met the inclusion criteria were randomly assigned to the amoxicillin group (*n* = 50) and the placebo group (*n* = 50). The final study sample consisted of 96 patients, as 3 patients in the amoxicillin group were lost to follow-up: 2 because they did not attend the check-ups and 1 because they did not follow the study instructions correctly. In the placebo group, one patient was lost due to non-attendance at follow-up appointments. Figure 1 depicts the flow of patients through the study.

As shown in Table 2, the mean age of the patients was 57.09 ± 11.48, 49 patients (51%) were male and 47 patients (49%) were female; 61 patients (63.5%) were ASA I and 35 patients (36.5%) ASA II; 28 patients (29.2%) were taking systemic medication and 68 patients (70.8%) were not, 12 patients (12.5%) were smokers and 84 (87.5%) were non-smokers, 52 patients (54.2%) had controlled periodontal disease and 44 patients (45.8%) had no periodontal disease. In terms of location 64 patients (66.7%) received implants in the maxilla and 32 patients (33.3%) in the mandible. Fifty-nine patients (61.5%) received 1 implant, thirty-two patients (33.3%) received 2–3 implants and only five patients (5.2%) received 4–6 implants; the total mean number of implants per patient was 1.57 ± 1.02. Most implants were placed in patients with partial edentulism; 89 (92.71%). A linear incision was used for the majority of procedures, 89 (92.71%), and the mean surgery time was 38.25 ± 19.91 min. None of the patients in the study groups reported any adverse effects from the medication.

All variables were homogeneously distributed between the two study groups, with no statistically significant differences (*p* > 0.05) except for surgery time, which was longer in the antibiotic group than in the placebo group (*p* = 0.049).

### 3.2. Early Implant Failure

A total of 151 implants were placed in 96 patients, 78 implants in the amoxicillin group and 73 implants in the placebo group. Twelve implants failed in 11 patients. Regarding the implants (Table 3), out of the 151 implants a total of 12 implants failed, representing an overall implant survival rate of 92.1%; 6 of them were lost in the amoxicillin group (7.7%) and 6 in the placebo group (8.2%), so there was no statistically significant difference between the two study groups regarding early implant failure (*p* = 0.911). In the amoxicillin group, 1 implant was lost at 7 days, 1 at 30 days and 4 at 90 days, while in the placebo group, 1 was lost at 14 days, 3 at 30 days and 2 at 90 days.

When analyzing the influence on early implant failure of the patient, implant and intervention variables collected, no association of any of these variables with early failure was found (*p* > 0.05) (Table 4).

In contrast, patients with higher pain values from postoperative day 2 to 7 and greater swelling from day 4 to 6 did have a higher incidence of early implant failure (*p* < 0.05) (Table 5).

### 3.3. Infectious Complications

Postoperative infection was recorded in 11 implants (Table 3), 2 of them in the amoxicillin group (2.6%) and 9 in the placebo group (12.3%), reaching statistically significant differences between the two study groups, such that implants placed in patients in the placebo group presented more infectious complications than those in the amoxicillin group (*p* = 0.041).

Statistical analysis shows a significant increase in the risk of infection in the experimental group that did not receive antibiotics compared to the group that did receive them. Specifically, the odds ratio (OR) for infection, when comparing the ‘no antibiotics’ group with the ‘antibiotics’ group, was 5.344 (95% Wald CI: 1.075–26.567).

Specifically, the Odds Ratio (OR) for infection, when comparing the “no antibiotic” group to the “with antibiotic” group, was 5.344 (Wald IC 95%: 1.075, 26.567). The majority of infectious complications in both the amoxicillin group (100%) and the placebo group (66.7%) occurred within the first 7 days. Of the 11 implants with postoperative infection, 3 (27.3%) of them eventually failed: 1 in the antibiotic group with infection at 7 days and 2 in the placebo group with infection at 7 days.

When we related the variables early failure and postoperative infection, we found that the presence of postoperative infection in the first 7 days, represented by very severe pain after the first 48–72 h after implant placement, was associated with a higher risk of early implant failure (*p* = 0.017) (Table 6). A statistically significant association was observed between the absence of severe postoperative pain and the probability of implant failure. Specifically, the ‘Severe Pain’ group exhibited an Odds of implant failure approximately 11.76 times higher than the ‘No Severe Pain’ group. The 95% Confidence Interval (CI) for this Odds Ratio (OR) of 11.76 (representing the Odds of failure for the ‘Severe Pain’ group compared to the ‘No Severe Pain’ group) was estimated to be [1.304, 111.111].

## 4. Discussion

This clinical trial compares the influence of an antibiotic prophylaxis of 2 g amoxicillin 1 h before surgery versus placebo in ASA I and ASA II patients undergoing dental implant placement in a single operative field, finding no differences between the two groups in relation to early implant failure. On the other hand, a greater number of infectious complications have been found in patients who do not receive antibiotics, with a 4.8 times higher risk of developing an infection compared to the antibiotic group. Furthermore, when this infection occurs in the first 7 days, the probability of implant failure is higher.

Rodriguez-Sanchez et al. [20] have already observed that single-dose oral amoxicillin preoperatively (SDOAP) is beneficial when compared to postoperative oral amoxicillin. Only SDOAP is effective and efficacious at preventing implant failures, but it was not significant for postoperative infections following dental implant surgeries. These results are consistent with the present RCT. In addition, these findings were confirmed in the trial by Tabrizi et al. [21], and in the systematic review and meta-analysis by Tan et al. [22], in which they conclude that a preoperative regimen of 2 g amoxicillin significantly reduces early implant failure in healthy subjects. Khouly et al. [23], in their review and meta-analysis, found no statistically significant differences in infection after surgery between those taking antibiotics and those taking a placebo. Torof et al. [24] also analyzed the NNT (Number Needed to Treat) to prevent one implant failure, which was 14, i.e., antibiotics had to be administered to 14 patients to prevent one implant failure caused by infection. Momand et al. [25] published the most recent review in this area, in which the sample size is smaller (1859 patients) than the 2023 review by Torof et al. [24] (2951 patients), justifying that the inclusion criteria used were stricter. In this systematic review, Momand et al. [25] found no significant difference between the use of antibiotic prophylaxis or placebo to reduce implant failure, obtaining an NNT of 143. Since these values are higher than 5, it indicates that the intervention is not efficient enough to be used routinely and that, therefore, each case should be individualized. On the other hand, in cases where implants are placed simultaneously with bone regeneration or in immediate post-extraction implants, a previous regimen of 2 or 3 g of amoxicillin is recommended [26], and in the case of immediate implants, continue the regimen of 500 mg amoxicillin 1 every 8 h for 5–7 days in order to prevent infection [27].

Despite numerous studies, the use of antibiotic prophylaxis for dental implant procedures in healthy patients is still under debate. Systematic reviews advise its use in implant placement to prevent early failure and infectious complications [9,28,29,30], while other more recent clinical trials and reviews state that the use of antibiotics in healthy patients for prophylaxis of surgical infection and early failure associated with dental implant placement does not seem to improve clinical outcomes and that practitioners should apply the principles of antimicrobial stewardship and not use antibiotics [19,23,31], thus the 2024 systematic reviews by Mehrabanian et al. [32] and Momand et al. [25] conclude, as we do, that routine antibiotic prophylaxis does not reduce the risk of early implant failure in healthy patients and that given the limited benefit and concern about increasing antibiotic resistance, their systematic use in dental implant surgery should be avoided.

Some studies and systematic reviews have analyzed the efficacy of certain local antibiotics [33,34] and other compounds such as PRF [35] in oral surgery and implant dentistry. In this sense Olvera-Huertas et al. [33] showed that osteoblast growth and differentiation may be favored by low doses of clindamycin, and it may be the decontaminant of choice for intraoral bone grafts, while CHX is shown as a less bone-friendly agent. In addition, Niemczyk et al. [35] showed that antibiotic-loaded PRF carriers are a potent tool for localized antimicrobial delivery, with promising applications in clinical settings.

Well-defined clinical assessment pathways similar to those used for other medical conditions are needed, taking into account the patient’s age, dental risk factors (such as oral and bone health), physical risk factors (such as chronic or long-term diseases) and modifiable health determinants (such as smoking). This is key to avoiding unnecessary antibiotic use.

In the recent clinical trial by Majid [17], the interventions were also performed by inexperienced operators (postgraduate implant students) and in patients with more than 1 implant (2–4 implants) but with an antibiotic prophylaxis regimen of only 1 g amoxicillin and unlike our study, if they find a significantly higher rate of implant loss in the placebo group (14.9%) compared to the antibiotic group (2.3%) and advise the prophylactic administration of antibiotics after implant surgery performed by inexperienced practitioners, these results may be justified by the lower dose of preoperative antibiotic used. On the other hand, and in contrast to our results, they do not report a higher incidence of signs of infection after 7 days.

On the other hand, the rate of early implant failure in our clinical trial was higher (7.9%) than most previous placebo-controlled studies, which report a frequency of 1.3% to 5.1% [22,36,37]. This is probably because we have included in our sample patients who may have a higher risk of early implant failure such as smokers, patients with periodontal disease (although always treated), implants performed after atraumatic sinus lift prior to implant placement and also because most clinical trials are performed on single implants and not on multiple implants like our study [38,39,40], as our aim was to include healthy individuals but more representative of the usual patients receiving dental implants. However, it is true that when we analyzed the influence of all these higher-risk variables, such as smoking, periodontal disease, number of implants and atraumatic sinus lift techniques, we found no association with a higher incidence of early failure; only short implants, although without reaching significant differences, seem to be associated with a higher incidence of implant failure. Perhaps this higher incidence of implant failure than most studies can be justified more by the fact that the interventions were performed by inexperienced hands, as in the study by Majid [17], where the implants were placed by inexperienced practitioners, who also found a higher failure rate similar to ours, namely 8.6%.

Although antibiotic prophylaxis has been used for many years to prevent early implant failure and infectious complications [8,11], the current trend is to dispense with it for implant surgery in systemically healthy patients [24,25], due to studies such as that of Khalil et al. [41] who demonstrated that a single dose of 2 g of amoxicillin could alter the ecology of the oral microflora, leading to the appearance of resistant strains. And we already know that nowadays the problem of antibiotic resistance is becoming increasingly serious worldwide due to uncontrolled prescribing of antibiotics, which makes it very important to limit their use to strictly necessary clinical situations and to the correct doses and timing.

Most of the current studies include single implant placement and ASA I patients, so we considered it important to extend the sample to patients with single and multiple implants placed in a single operative field and also to ASA II patients.

The main limitation of our study design is the statistical power (1 − β) fixed at the 80% threshold for the prespecified clinically relevant minimum difference of 6 percentage points. Although this is the conventionally accepted minimum in clinical trial design, it implies a 20% risk of committing a Type II error (i.e., failing to reject the null hypothesis) if the true absolute difference in implant failure rates is less than 6 percentage points (e.g., 4 percentage points). Consequently, the study may be underpowered to detect smaller-magnitude effects, should they exist. This statistical compromise was necessitated by the need to maintain study feasibility given the low event rate and the logistical challenges inherent in patient accrual within a single-center setting. Future multicenter investigations with larger cohorts would be required to precisely quantify smaller differences.

Another methodological limitation of our study is the reliance on bivariate Generalized Estimating Equation (GEE) models. While the GEE approach correctly accounts for the clustering of implants within patients, the exclusive use of bivariate models means that potential confounding factors were not statistically controlled for in the final analysis. Although the randomized design of the trial aimed to distribute these factors equally between the treatment groups, the reported Odds Ratios (OR) for the antibiotic intervention are unadjusted and may therefore be susceptible to residual confounding. Consequently, the interpretation of the isolated effect of the antibiotic must be considered within the context of this lack of adjustment for other known risk factors influencing implant survival.

For future research, we believe that it would be interesting not only to increase the sample size but also to introduce other more current issues to be analyzed, such as the evolution of the microbiome in implants and neighboring teeth and the change in the marginal bone level between the radiograph of implant placement and the time of prosthetic loading, which could determine late implant failure depending on whether or not antibiotic prophylaxis is used.

## 5. Conclusions

The use of antibiotic prophylaxis to prevent early failure of implants placed in a single operative field does not seem to be justified, according to our results. However, in patients in whom antibiotics are not administered, the incidence of infectious complications increases and when occurring in the first 7 days, they are associated with a higher rate of early implant failure. This higher rate of infectious complications in patients without antibiotic therapy still raises a question that requires further research.

## Figures and Tables

**Table 1:** Inclusion and exclusion criteria.

Inclusion Criteria	Exclusion Criteria
-Patient ≥ 18 years of age	-Implants requiring simultaneous bone regeneration
-ASA I/ASA II	-Immediate post-extraction implants
-Need for dental implant placement	-Patients who require postoperative antibiotic prophylaxis due to their medical condition (e.g., immunocompromised, etc.)
-Periodontally controlled subjects	-Patients who have undergone head and neck radiotherapy less than two years ago
-Implants with sufficient bone and soft tissue that do not require augmentation surgery at the time of placement	-Uncontrolled diabetics
-Implants placed in a single surgical field	-Penicillin-allergic patients
	-Patients treated with antiresorptive drugs
	-Implants requiring simultaneous bone regeneration
	-Pregnant or breastfeeding women
	-Patients currently taking antibiotics for other reasons
	-Untreated periodontitis

**Table 2:** Patient and intervention characteristics comparing antibiotic prophylaxis group (*n* = 47) with placebo group (*n* = 49).

Variable		Amoxicillin Group (*n* = 47)	Placebo Group (*n* = 49)	*p* Value	Global (*n* = 96)
age		57.57 ± 11.15	56.63 ± 11.52	0.690	57.09 ± 11.48
sex	Male Female	27 (57.4%) 20 (42.6%)	22 (44.9%) 27 (55.1%)	0.229	49 (51%) 47 (49%)
general health	Asa I Asa II	32 (68.1%) 15 (31.9%)	29 (59.2%) 20 (40.8%)	0.402	61 (63.5%) 35 (36.5%)
systemic medication	Yes No	11 (23.4%) 36 (76.6%)	17 (34.7%) 32 (65.3%)	0.265	28 (29.2%) 68 (70.8%)
tobacco	Yes No	7 (14.9%) 40 (85.1%)	5 (10.2%) 44 (89.9%)	0.549	12 (12.5%) 84 (87.5%)
periodontitis	Yes No	29 (61.7%) 18 (38.3%)	23 (46.9%) 26 (53.1%)	0.158	52 (54.2%) 44 (45.8%)
implant localization	Maxilla Mandible	30 (63.8%) 17 (36.2%)	34 (69.4%) 15 (30.6%)	0.666	64 (66.7%) 32 (33.3%)
number of implant	1 2–3 4–6	24 (51.1%) 20 (42.6%) 3 (6.4%)	35 (71.4%) 12 (24.5%) 2 (4.1%)	0.122	59 (61.5%) 32 (33.3%) 5 (5.2%)
total number of implant		1.66 ± 0.96	1.49 ± 1.08	0.072	1.57 ± 1.02
edentulism	Partial Total	43 (91.5%) 4 (8.5%)	46 (93.9%) 3 (6.1%)	0.712	89 (92.7%) 7 (7.2%)
type of incision	Linear Linear with releasing incision	42 (89.4%) 5 (10.6%)	47 (95.9%) 2 (4.1%)	0.263	89 (92.7%) 7 (7.2%)
surgery duration		42.49 ± 22.55	33.9 ± 16.06	0.049 *	38.25 ± 19.91

* *p*-value obtained with the Mann–Whitney U test or *t*-Student test. Other *p*-values obtained with Fisher’s exact test or χ^2^ test.

**Table 3:** Comparison of early implant failure and postoperative infection during postoperative implant surgery follow-up (7, 14, 30, 90 days).

Outcome		Amoxicillin Group (*n* = 47)	Placebo Group (*n* = 49)	*p* Value	Global *n* = 96
implant failure	No Yes 7 days 14 days 30 days 90 days	72 (92.3%) 6 (7.7%) 1 (16.7%) 0 (0%) 1 (16.7%) 4 (66.7%)	67 (91.8%) 6 (8.2%) 0 (0%) 1 (16.7%) 3 (50%) 2 (33.3%)	0.911	139 (92.1%) 12 (7.9%)
postoperative infection	No Yes 7 days 14 days 30 days 90 days	76 (97.4%) 2 (2.6%) 2 (100%) 0 (0%) 0 (0%) 0 (0%)	64 (87%) 9 (12.3%) 6 (66.7%) 1 (11.1%) 2 (22.2%) 0 (0%)	0.041 *	140 (92.7%) 11 (7.3%)

* *p*-value obtained at the final period with GEE logistic regression model.

**Table 4:** Relationship of early failure to main patient, implant and intervention variables.

Outcome	Early Implant Failure		*p* Value
	Yes	No	
age (years) ≤55 56–65 >65	6 (12.2%) 3 (6.3%) 3 (5.6%)	43 (87.8%) 45 (93.8%) 51 (94.4%)	0.417
sex Male Female	7 (7.9%) 5 (8.1%)	82 (92.1%) 57 (91.9%)	0.966
systemic health ASA I ASA II	9 (9.1%) 3 (5.8%)	90 (90.9%) 49 (94.2%)	0.490
systemic medication Yes No	3 (8.3%) 9 (7.8%)	33 (91.7%) 106 (92.2%)	0.923
tobacco Yes No	0 (0.0%) 12 (8.9%)	16 (100%) 123 (91.1%)	-------
periodontitis Yes No	7 (8%) 5 (7.8%)	80 (92%) 59 (92.2%)	0.963
implant location Maxilla Mandible	9 (9.2%) 3 (5.7%)	89 (90.8%) 50 (94.3%)	0.539
number of implants 1 Implant 2–3 Implants ≥4 Implants	5 (8.5%) 6 (9.1%) 1 (3.8%)	54 (91.5%) 60 (90.9%) 25 (96.2%)	0.697
bone type Type I Type II Type III Type IV	0 (0%) 4 (7.1%) 6 (9.2%) 2 (7.7%)	4 (100%) 52 (92.9%) 59 (90.8%) 24 (92.3%)	0.924
implant length <8.5 mm ≥8.5 mm	4 (17.4%) 8 (6.3%)	19 (82.6%) 119 (93.7%)	0.101
implant diameter (mm) 3.3 mm 3.8–4.25 mm 5 mm	4 (11.4%) 8 (7.5%) 0 (0.0%)	31 (88.6%) 98 (92.5%) 10 (100%)	0.407
type of incision Linear Linear with releasing incision	11 (8.1%) 1 (6.7%)	125 (91.9%) 14 (93.3%)	0.823
bone expansion/condensation Yes No	1 (10%) 11 (7.8%)	9 (90.0%) 130 (92.2%)	0.805
biological drilling Yes No	4 (9.5%) 8 (7.3%)	38 (90.5%) 101 (92.7%)	0.668
atraumatic sinus elevation Yes No	1 (9.1%) 11 (7.9%)	10 (90.9%) 129 (92.1%)	0.875
SURGERY DURATION	51.67 ± 20.47	47.73 ± 24.22	0.555

GEE logistic regression model.

**Table 5:** Comparison of early implant failure and secondary outcomes during first week of follow-up (pain, inflammation and no pills).

	Early Implant Failure		*p* Value
	Yes	No	
day 1 Mean Pain Mean Inflammation	4.43 ± 3.46 3.00 ± 2.45	2.77 ± 2.22 1.96 ± 2.06	0.115 0.158
day 2 Mean Pain Mean Inflammation	4.71 ± 3.50 2.88 ± 2.75	1.59 ± 1.87 1.83 ± 1.97	0.001 * 0.160
day 3 Mean Pain Mean Inflammation	4.86 ± 3.48 2.63 ± 2.62	1.14 ± 1.85 1.45 ± 2.00	0.004 * 0.068
day 4 Mean Pain Mean Inflammation	4.14 ± 3.29 2.13 ± 1.46	0.97 ± 1.83 1.05 ± 1.75	0.005 * 0.009 *
day 5 Mean Pain Mean Inflammation	4.00 ± 3.61 1.75 ± 1.39	0.91 ± 1.93 0.63 ± 1.31	0.006 * 0.016 *
day 6 Mean Pain Mean Inflammation	2.86 ± 3.48 1.07 ± 1.00	0.59 ± 1.42 0.79 ± 0.00	0.115 * 0.058
day 7 Mean Pain Mean Inflammation	2.71 ± 3.55 0.63 ± 1.06	0.23 ± 0.63 0.27 ± 0.72	0.006 * 0.172
No Pills (7 days)	13.88 ± 11.09	6.94 ± 7.44	0.066

* *p*-value obtained with GEE logistic regression model.

**Table 6:** Infectious complications and early implant failure in 7 days.

Infectious Complications Variables	Early Implant Failure		*p* Value
	Yes	No	
Intense Pain (48–72 h) yes no	3 (37.5%) 9 (6.3%)	5 (62.5%) 134 (93.7%)	0.028 *
Inflammation since day 7 yes no	1 (20%) 11 (7.5%)	4 (80%) 135 (92.5%)	0.121
Fever yes no	---- 12 (7.9%)	---- 138 (92.1%)	----
Heat yes no	1 (100%) 12 (8%)	---- 138 (92%)	----
Suppuration (Fistula) yes no	---- 12 (8.1%)	2 (100%) 137 (91.9%)	----

* *p*-value obtained with GEE logistic regression model.

## Data Availability

The original contributions presented in this study are included in the article material. Further inquiries can be directed to the corresponding author.

## Supplementary Materials

The following supporting information can be downloaded at: https://www.mdpi.com/article/10.3390/dj13110500/s1, Table S1: CONSORT 2025 checklist item description.
